# Nucleoporin Nup58 localizes to centrosomes and mid-bodies during mitosis

**DOI:** 10.1186/s13008-019-0050-z

**Published:** 2019-08-03

**Authors:** Hartono, Masaharu Hazawa, Kee Siang Lim, Firli R. P. Dewi, Akiko Kobayashi, Richard W. Wong

**Affiliations:** 1 School of Natural System, Institute of Natural Science and Technology, Kanazawa University, Kanazawa, Japan; 2 Biology Department, Faculty of Mathematics and Natural Science, Universitas Negeri Makassar, Jl. Dg Tata Raya, Makassar, 90224 Indonesia; 3 Cell-Bionomics Research Unit, INFINITI, Kanazawa University, Kanazawa, Japan; 4 WPI-Nano Life Science Institute, Kanazawa University, Kakuma-machi, Kanazawa, Japan; 5 Biology Department, Faculty of Science and Technology, University of Airlangga, Jl. Airlangga No. 2-4, Gubeng, Surabaya, East Java 60115 Indonesia

**Keywords:** Nup58, Centrosome, Midbody, Mitosis, Cytokinesis

## Abstract

**Background:**

Nuclear pore complexes (NPCs) act as nano-turnstiles within nuclear membranes between the cytoplasm and nucleus of mammalian cells. NPC proteins, called nucleoporins (Nups), mediate trafficking of proteins and RNA into and out of the nucleus, and are involved in a variety of mitotic processes. We previously reported that Nup62 localizes to the centrosome and mitotic spindle during mitosis, and plays a role in centrosome homeostasis. However, whether Nup58, a Nup62 subcomplex protein, also localizes to spindle poles is unknown.

**Result:**

Herein, we show that Nup58 localizes to the nuclear rim during interphase, and to mitotic spindles, centrosomes, and midbodies during mitosis. Our confocal microscopy, live-cell imaging, and stimulated emission depletion nanoscopy results also demonstrated that Nup58 localized to the centrosomes during metaphase and relocalized to midbodies during abscission. Depletion of Nup58 resulted in centrosomal abnormalities and delayed abscission.

**Conclusion:**

Nup58 localized at the centrosomes and mitotic spindle during metaphase and relocalized at midbodies during abscission. This study highlights the important role of Nup58 in mitosis.

**Electronic supplementary material:**

The online version of this article (10.1186/s13008-019-0050-z) contains supplementary material, which is available to authorized users.

## Background

Nuclear pore complexes (NPCs) control trafficking of macromolecules between the cytoplasm and nucleus in interphase cells [1–3]. NPCs consist of approximately 30 proteins (called nucleoporins/Nups), which form a central nano-turnstile with filaments extending into both the nucleus and cytoplasm [4, 5]. Macromolecular transport across the pore occurs rapidly by an energy-dependent process. Nuclear trafficking pathways are regulated by an intracellular gradient of the small GTPase Ran, with a high concentration of RanGTP in the nucleus and high concentration of RanGDP in the cytoplasm. In addition to macromolecular trafficking, Nups also contribute to the control of gene expression, chromatin maintenance, and mitotic progression [6, 7]. Indeed, as mammalian cells enter mitosis, the nuclear envelope pulls apart during its breakdown process, which involves the disassembly of NPCs and nuclear lamina, as well as release of the nuclear envelope membrane from chromatin [8, 9]. An increasing number of Nups have now been demonstrated to function at kinetochores, centrosomes, and spindles during mitosis [10–13]. NPC components are actively involved at various stage during mitosis. However, precise roles of individual Nups, both spatially and temporally, during cell cycle progression are still largely unknown (for review see: [14–17]). Using conventional confocal microscopy and live-cell imaging techniques, we previously demonstrated the mitotic functions [16] of nucleoporin Rae1 [18–21], Nup88 [22], Tpr [23–26], and Nup358 [27]; more recently, we also revealed that Nup62 plays a novel role in centrosome integrity during mitosis [28, 29].

Nup62 is a constituent of the Nup62 complex that also includes Nup58 and Nup54 [30, 31], which are anchored by Nup93 at the pore and are critical for NPC function [32]. Although Nup62 localizes to the centrosome during mitosis, little is known about the spatiotemporal localization and functions of Nup58 [33, 34]. Depletion of Nup58 by RNA interference (RNAi) has been shown to reduce viability of colorectal cancer cell lines (DLD1 and SW480) [35]. Moreover, knockdown of Nup58 by RNAi in MDCK cell lines elicited cell polarity defects [36]. Further, Nup58 also facilitates metastasis and epithelial-mesenchymal transition (EMT) of lung adenocarcinoma via the GSK-3β/Snail signaling pathway during interphase [37]. However, the functional consequences of Nup58 in the cell cycle of cancer cells remains unknown.

In this report, we address whether Nup58 plays a role in regulating cell cycle progression. We demonstrate that Nup58 localized to the centrosomes and midbody at the metaphase-telophase-cytokinesis transition during cell division. In addition, Nup58 depletion further influenced the abscission process, resulting in defective cytokinesis.

## Results

### Nup58 localized to mitotic spindles and spindle poles during mitosis

**Fig. 1:**
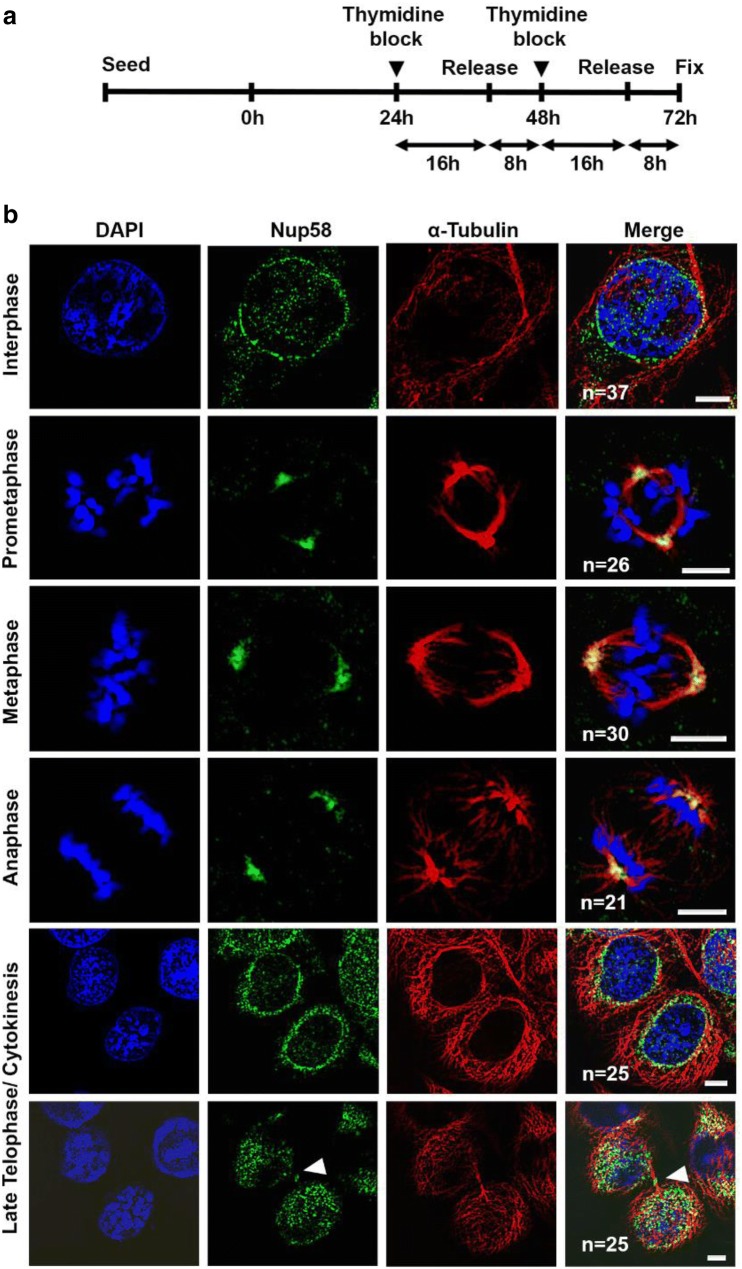
Localization of Nup58 on mitotic spindles during mitosis, as acquired with a confocal microscope. **a** Schedule of collecting mitotic HeLa cells for confocal microscopy imaging. **b** Deconvoluted confocal images of HeLa cells at different cell cycle stages. Green, anti-Nup58; red, anti-α-tubulin; blue, chromatin (DAPI). Scale bars, 5 µm

To further clarify specific roles of Nup58 during mitosis, we sought to investigate the detailed subcellular localization of Nup58 during the cell cycle in HeLa cells. We first synchronized HeLa cells using a double-thymidine block (Fig. 1a) and examined Nup58 localization during cell division. HeLa cells were coimmunostained for endogenous Nup58 and α-tubulin. We observed dynamic localization of Nup58 within different mitotic and cytokinetic subcompartments. During interphase, Nup58 was present in the cytoplasm and on the nuclear envelope, thereby forming a typical nuclear rim pattern in HeLa cells (Fig. 1b). However, during prometaphase and metaphase, Nup58 gradually accumulated into spindle-like structures, which were identified by co-staining with an α-tubulin antibody; Nup58 was also detectable at mitotic spindle poles or centrosomal regions during metaphase. As cells progressed into late telophase or cytokinesis, Nup58 was no longer detectable at the centrosome, but instead ephemerally localized at the midzone (Fig. 1b, Additional file 1: Fig. S1). Thus, our immunofluorescence data indicated that Nup58 has dynamic localization during the cell cycle and is associated with the cytokinesis apparatus during the late telophase-cytokinesis transition.

### Nup58 localized to centrosomes during the metaphase–anaphase transition

**Fig. 2:**
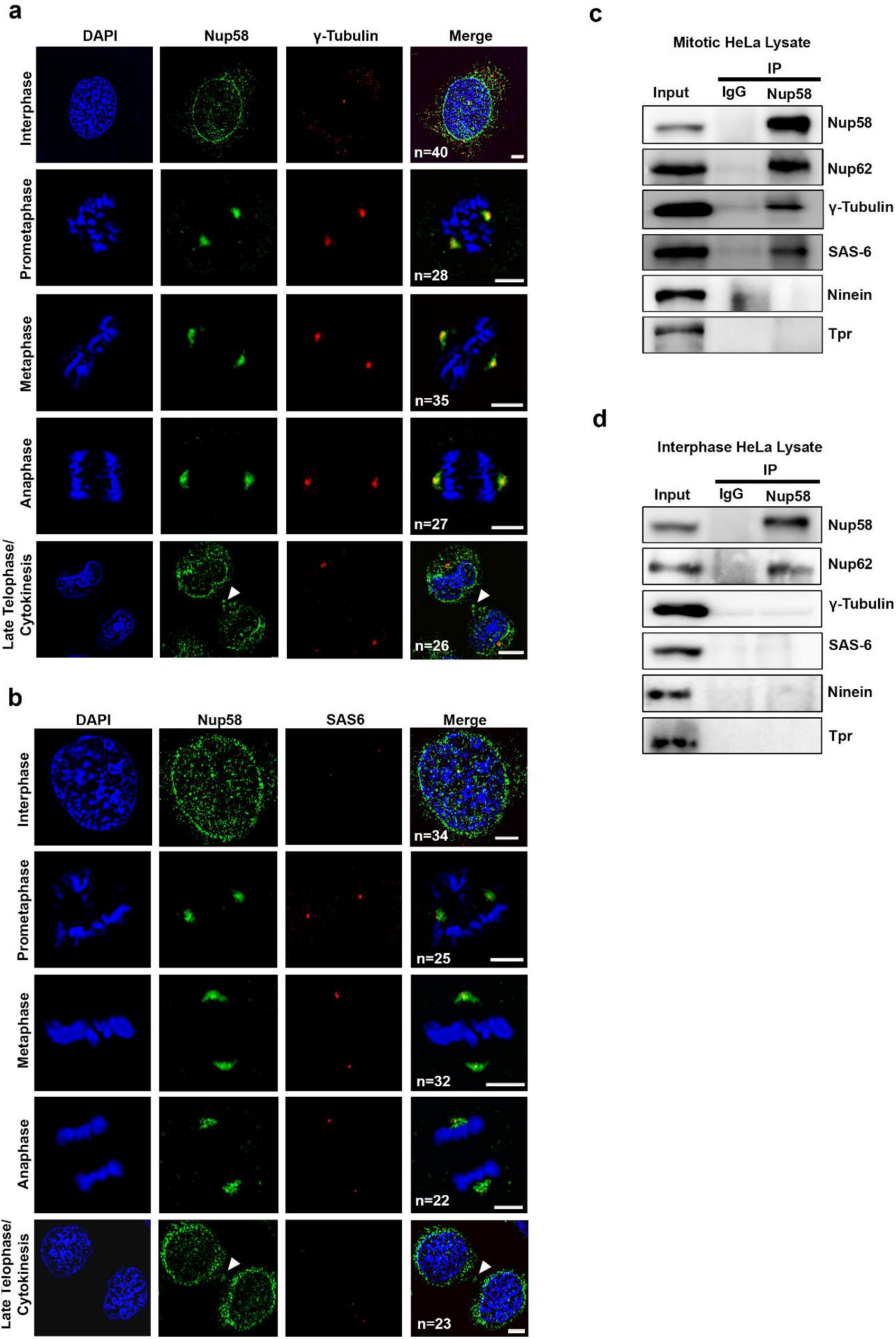
Localization of Nup58 on the centrosome and its interaction with centrosome protein during cell division. **a**, **b** Deconvoluted confocal images of HeLa cells at different stages in the cell cycle. Green, anti-Nup58; red, anti-γ-tubulin (**a**) or anti-SAS-6 (**b**); blue, chromatin (DAPI). Scale bars, 5 µm. **c**, **d** Immunoprecipitation of HeLa cell extracts with nonspecific rabbit antibodies (IgG) or anti-Nup58, as analyzed by SDS-PAGE and immunoblotting with antibodies against Nup58, Nup62, γ-tubulin, SAS-6, ninein, and Tpr during mitosis (**c**) and interphase (**d**)

The metaphase observations described above led us to surmise that Nup58 contributes to centrosome assembly during cell division. Indeed, we showed that a fraction of Nup62, the *bona fide* binding partner of Nup58, localized to centrosomes during mitosis [28]. To investigate whether Nup58 also localized to centrosomes, we used specific antibodies against centrosome markers Nup58, γ-tubulin, and SAS-6 to examine their localization at different cell cycle stages. Deconvoluted confocal imaging of HeLa cells indicated colocalization of Nup58, SAS-6, and γ-tubulin (Fig. 2a, b). Moreover, a series of maximum projections of the z-plane were captured to rule out potential staining artifacts (Additional file 1: Fig. S2a-c). Given the colocalization of Nup58 with γ-tubulin at the centrosome, we sought to investigate whether Nup58 physically interacts with centrosomal proteins. Thus, we next examined whether Nup58 was associated with centrosomal proteins in HeLa cells during interphase or mitosis. Using immunoblotting of anti-Nup58 immunoprecipitates, we detected coprecipitating γ-tubulin and SAS-6, but not the coiled-coil centrosomal protein ninein during mitosis (Fig. 2c). To confirm the binding specificity and sensitivity of Nup58 in our immunoprecipitation assay, we used ninein as negative control because it has a major role in microtubule minus-end anchorage and also acts as a docking site for γ-tubulin-containing complexes at centrosomes [38]. Similarly, Tpr, which acts as another negative control Nup, localized to the nuclear basket region [25]. Moreover, we found that Nup58 did not pull down any centrosomal proteins during interphase (Fig. 2d). These data suggest that Nup58, along with Nup62, interact with γ-tubulin and SAS-6 at the spindle pole/centrosome during mitosis. Thus, our staining data indicated that endogenous Nup58 localized at centrosomal regions during the metaphase–anaphase transition, although the biological function of the interaction between Nup58 and centrosome markers during cell division needs further exploration.

### Nup58 localized to the dark zone region of midbodies during telophase

**Fig. 3:**
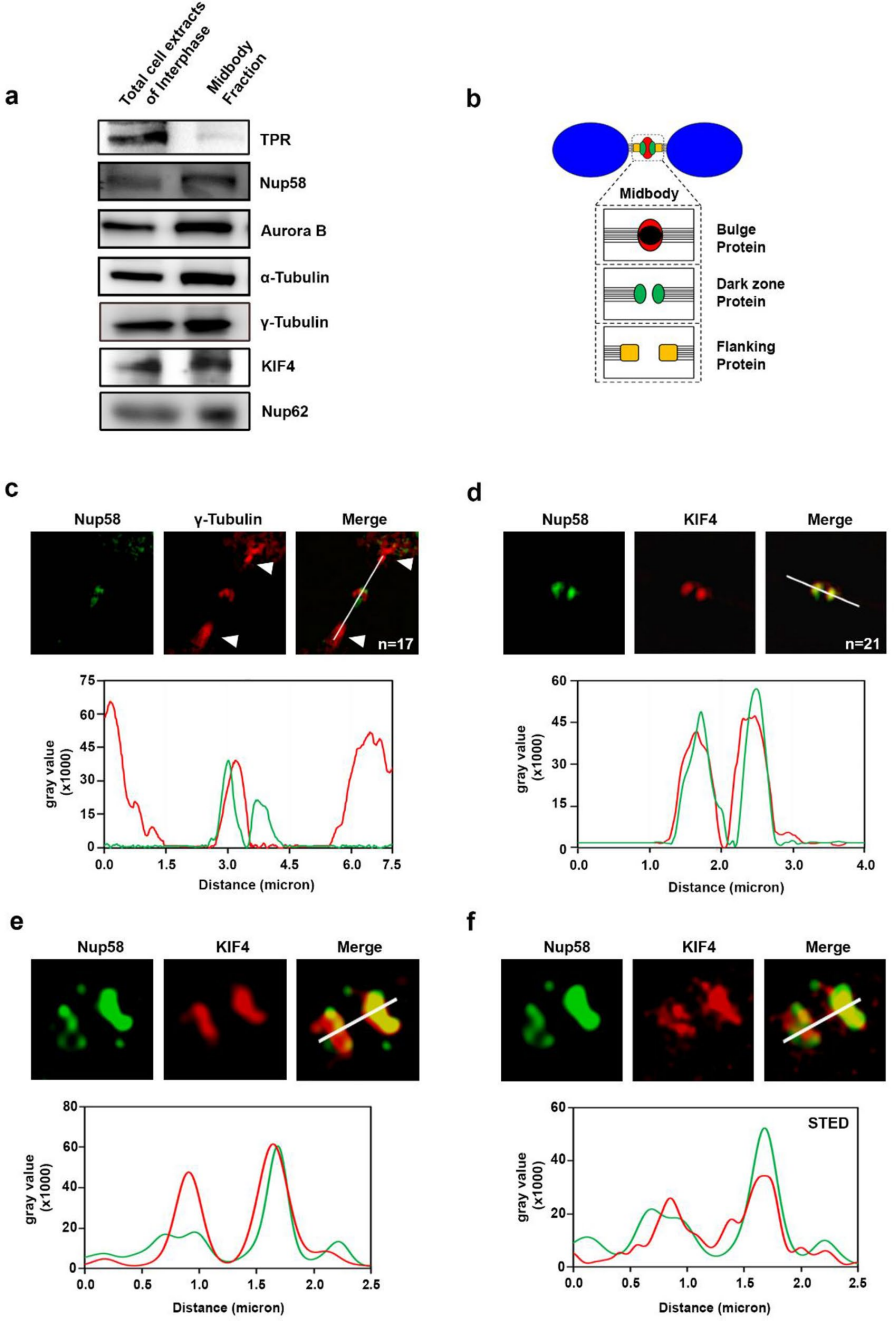
Colocalization of Nup58 with midbody protein markers. **a** Total cell extracts of interphase cells and extracts of purified midbodies were examined by immunoblotting with the indicated antibodies. **b** Categorization of midbody proteins according to localization on the midbody. **c**, **d** Deconvoluted confocal images of HeLa cells during stages of cytokinesis. **e**, **f** Super-resolution images of Nup58 and KIF4 acquired with confocal and STED microscopy, respectively. Green, anti-Nup58 ab; red, anti-γ-tubulin (**c**) or anti-KIF4 (**d**–**f**). Line scans show the positions of proteins in relation to each other

We next isolated and purified midbody extracts and confirmed that Nup58 and Nup62 were indeed accumulated in the biochemical midbody fraction. Moreover, to confirm the specificity of fractionation, we blotted Tpr as a negative control and Aurora B, α-tubulin, γ-tubulin, and KIF4 as positive controls (Fig. 3a). Midbody protein localization was further categorized into two main groups: bulge and dark zone-flanking regions (Fig. 3b). We compared Nup58 midbody localization by counterstaining for two different midbody marker proteins, such as γ-tubulin (bulge protein marker) and KIF4 (dark zone protein marker) (Fig. 3b, Additional file 1: Fig. S1). To further refine and validate Nup58 midbody localization, we performed stimulated emission depletion (STED) nanoscopy. We co-stained for Nup58 and KIF4 in mitotic HeLa cells and found that KIF4 associated with Nup58 at midbodies, consistent with immunofluorescence results. In addition, our STED data also revealed that mitotic Nup58 completely colocalized with KIF4 at the dark zone-flanking region, but not at the bulge (with γ-tubulin) at midbodies during cytokinesis (Fig. 3c–f). Altogether, during the telophase-cytokinesis transition, Nup58 signal was detected in two disks on either side of the midbody. These data suggest that Nup58 first associates with the centrosome in metaphase, and remains accumulated at the finest portions surrounding the dark-flanking zone in the midbody during abscission.

### Nup58 relocalized to the midbody during telophase

**Fig. 4:**
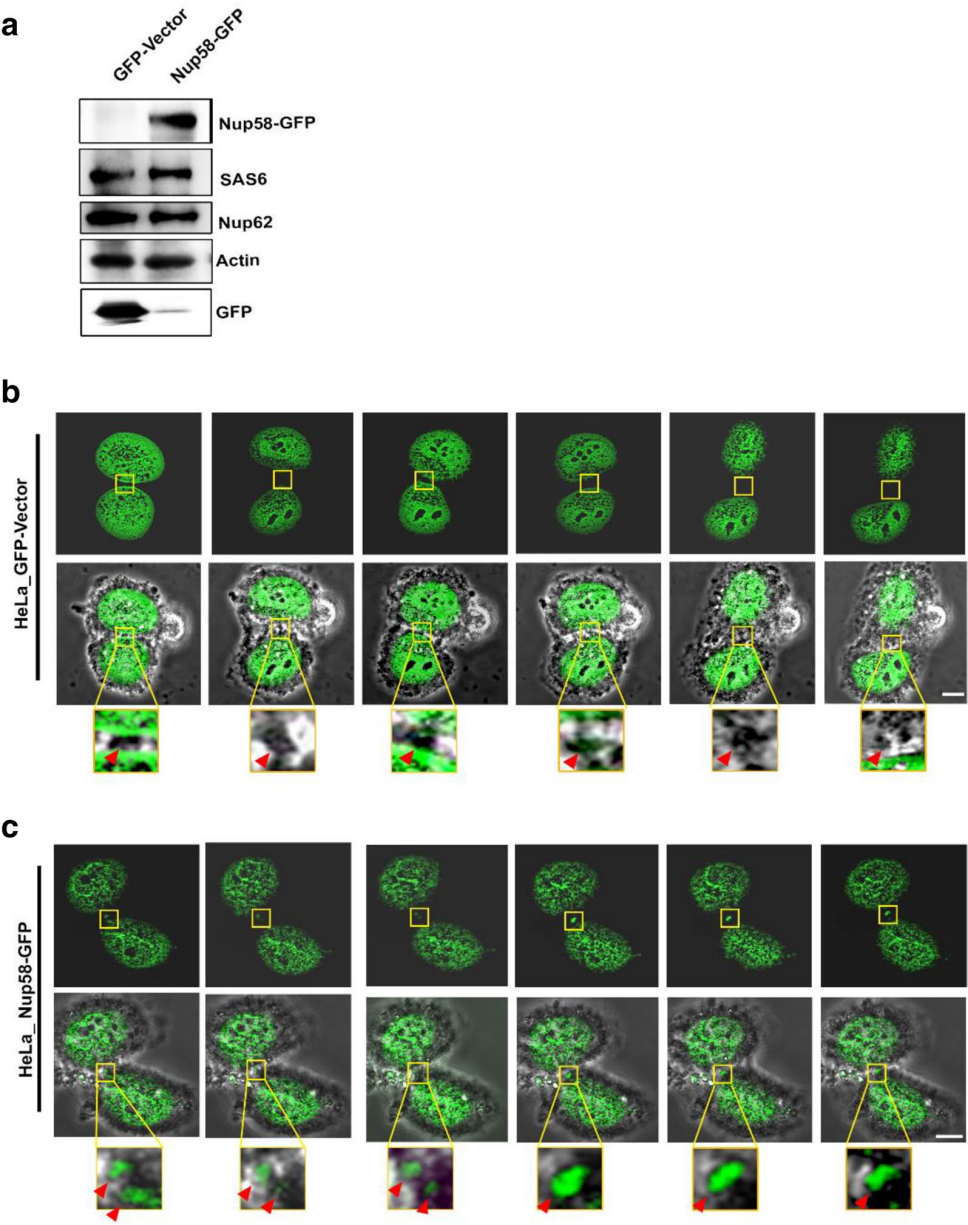
Localization of Nup58 on the midbody during cytokinesis. **a** Cell lysates of HeLa cells transfected with Nup58–GFP expression plasmid or GFP-vector were analyzed by immunoblotting for GFP or Nup58–GFP. **b** Live-cell images of GFP-vector-expressing HeLa cells during cytokinesis from midbody maturation to final abscission (Additional file 3: Video 2). **c** Live-cell images of Nup58–GFP-expressing HeLa cells during cytokinesis from midbody maturation to final abscission showing localization of Nup58 on the midbody. Insets are magnifications of midbody areas in observed cells (Additional files 4, 5: Video 3a, 3b). Arrowheads indicate HeLa cell midbodies during cytokinesis. Scale bars, 5 µm

To prevent non-specificity in the expression pattern established using a Nup58 antibody and further ratify the centrosomal or midbody-like localization of Nup58 during mitosis, we performed real-time live imaging of Nup58. An N-terminal green fluorescent protein (GFP)-tagged Nup58 was generated to detect expression of the GFP-tag (Fig. 4a). Live-cell imaging analysis was used to determine Nup58-GFP cell cycle localization. A representative series from time-lapse images of GFP-Nup58 during interphase–anaphase transitions is shown in Additional file 2: Video 1. We observed a GFP-Nup58 staining pattern around the nuclear rim during interphase, and then at the centrosome during metaphase, which dynamically relocalized back to the newly developed chromatin boundary during late anaphase (Additional file 2: Video 1). Consistent with immunofluorescence data (Figs. 1, 2 and 3, Additional file 1: Fig. S1), we also found that some GFP-Nup58 partially localized to midbody regions during late telophase and cytokinesis (Fig. 4b, c; Additional files 4, 5: Video 3a, 3b). Notably, with live-cell imaging, we found that Nup58-GFP signals were obviously detectable at the midbody, where they remained until the abscission process finished [Fig. 4c (red arrowheads) and Additional files 4, 5: Video 3a, 3b] compared with GFP vector alone (Fig. 4b, Additional file 3: Video 2). Thus, our live-cell imaging data suggest that, in addition to its well-defined nuclear envelope localization, Nup58 also partially relocalizes at the centrosome during metaphase and midbody during abscission.

### Nup58 depletion triggered centrosomal abnormalities and delayed abscission

**Fig. 5:**
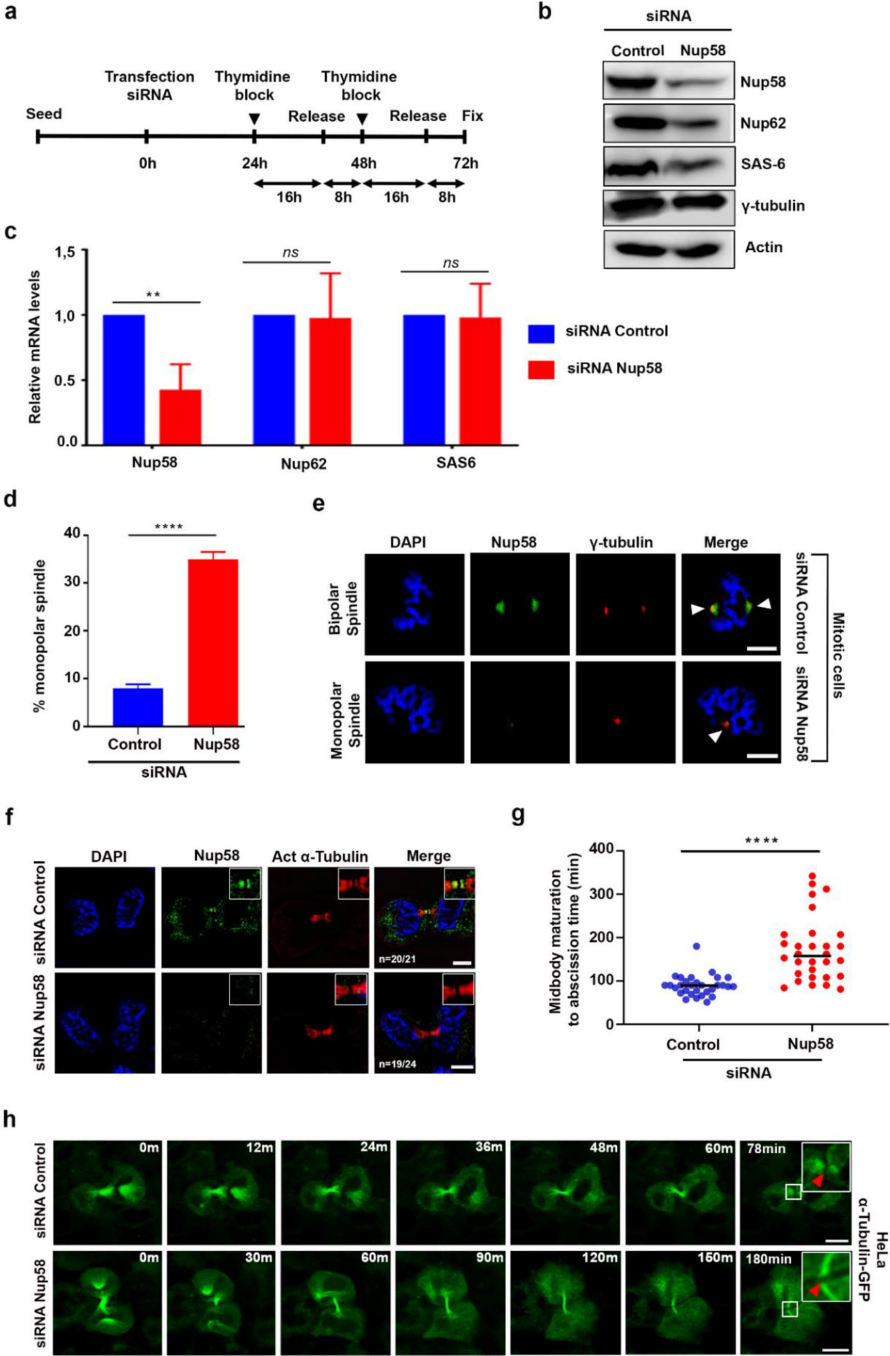
Nup58 depletion induced centrosomal abnormalities and delayed cytokinesis. **a** Brief agenda of collecting cells after Nup58 knockdown via siRNA transfection. **b** HeLa cells were transfected with control or Nup58 siRNA, then cell lysates were collected 72 h after transfection and analyzed by immunoblot of Nup58, Nup62, SAS-6, γ-tubulin, and β-actin expression. **c** qRTPCR analysis of Nup58, Nup62, and SAS-6 mRNA in HeLa cells after transfection with siRNA Nup58 for 72 h. Data show mean ± SD of three separate experiments. Significance difference was assessed with a Student’s t-test, **p < 0.01. **d** Quantification (relative %) of abnormal centrosome phenotypes in control or Nup58 siRNA-transfected HeLa cells. Values are based on three independent experiments (*n *= 500 cells). Mean values ± SD (error bars) are shown. Significance difference was assessed with a Student’s t-test, ****p < 0.0001. **e** Deconvoluted confocal images of synchronous HeLa cells, transfected with control or Nup58 siRNA, 72-h post-transfection. Green, anti-Nup58; red, anti-α-tubulin; blue, chromatin (DAPI). Scale bars, 5 µm. White arrows indicate bipolar and monopolar spindles as centrosome abnormalities when Nup58 was completely depleted. **f** Deconvoluted confocal images of HeLa cells 72-h post-transfection with control or Nup58 siRNA. Green, anti-Nup58; red, anti-acetylated-γ-tubulin; blue, chromatin (DAPI). Scale bars, 5 µm. Insets are magnifications of the midbody areas in observed cells. **g** Midbody maturation to abscission time in cells transfected with Nup58 siRNA (*n *= 30 cells) compared with control (*n *= 30 cells). Mean values ± SD (error bars) are shown, and significant differences were assessed with a Mann–Whitney U test, ****p < 0.0001. **h** Confocal live-cell images of stable GFP-α-tubulin expression in HeLa cells transfected with Nup58 siRNA shows delayed cytokinesis expression compared with the control. Insets are magnifications of microtubules areas in observed cells (Additional file 6: Video 4). Arrowheads indicate microtubules abscission. Scale bars, 5 µm

To further analyze the mitotic role of Nup58, we used a Nup58 siRNA-knockdown approach (Fig. 5a). Immunoblotting analysis of HeLa cells subjected to Nup58 siRNA treatment for 3 days revealed ~ 77% reduction in Nup58 compared with controls (Fig. 5b, Additional file 1: Fig. S3). Moreover, there were no significant changes in γ-tubulin levels. Notably, Nup62 and SAS-6 levels were reduced compared with mock-treated (control siRNA) cells (Fig. 5b). Moreover, we did not observe significant changes in mRNA levels of Nup62 or SAS-6 by qPCR, apart from potential Nup58 siRNA off-target effects (Fig. 5c). To confirm that mitotic phenotypes were specific to Nup58 and not a secondary effect arising from other problems with nuclear transport, we also examined the level of importin-β, a major protein in nuclear transport, in cytoplasmic and nuclear fractions of Nup58-knockdown cells and controls. Our results revealed that there was no significant difference in protein levels in either cell type (Additional file 1: Fig. S4). This result is in line with a study by Rodriguez-Berriguete et al. [39] reporting that knockdown of Nup58 does not impair nuclear import. Furthermore, we found that centrosomal abnormalities were increased three-fold compared with control siRNA mitotic cells (*n* = 500 mitotic cells). In contrast with centrosomal abnormalities induced by Nup62 knockdown, which mostly involve the formation of multipolar spindles [28], Nup58 depletion induced monopolar spindles including spindles with poorly separated poles (34.96% vs. 7.93%; *p* < 0.0001; Fig. 5d). Moreover, compared with the staining pattern in control siRNA cells, downregulation of Nup58 also induced a marked ~ 25% increase in the formation of monopolar spindle (co-staining with γ-tubulin, as a centrosome marker, *n* = 500 mitotic cells) (Fig. 5d, e). Next, we analyzed cytokinesis in Nup58-depleted cells in detail. In fixed-cell images, siNup58-treated cells exhibited a considerably shifted distribution of midbody widths together with elongated and thin midbodies (Fig. 5f, g). Midbody maturation to abscission was also significantly slower when Nup58 was depleted (Fig. 5g, *p *< 0.01). On the basis of observed late-stage midbody defects, we further examined whether loss of Nup58 induced delays or failures in abscission by carrying out live-cell time-lapse imaging with siRNA-treated HeLa cells stably expressing GFP-α-tubulin. Figure 5h shows a typical example of time-lapse images of abscission collected via confocal microscopy (Fig. 5h, Additional file 6: Video 4). We recorded significant delays in the abscission process (> 180 min) in Nup58-depleted cells compared with control siRNA mitotic cells (~ 70 min; Fig. 5h, Additional file 6: Video 4). Further, in response to Nup58 depletion, cell division was halted primarily in metaphase, and those Nup58-depleted monopolar spindle cells exhibited severe mitotic catastrophe and cell death (Additional file 7: Video 5).

## Discussion

In recent years, Nups have been shown to perform a great variety of alternative functions, many of which are apparently unrelated to nuclear transport [2, 4, 7]. Herein, we found evidence for an additional role of Nup58, a NPC member required for nuclear pore biogenesis [3], in cytokinesis; namely, temporal regulation of telophase, cytokinesis, and abscission. Using live-cell imaging and STED nanoscopy, we revealed that Nup58 transiently localized to the centrosomes (Additional file 2: Video 1) and midbody (Additional file 4, 5: Video 3a, 3b), and was especially enriched near the midbody core and around dark zone/flanking regions; these patterns are distinct from other Nups.

Indeed, during cytokinesis, contraction of the actomyosin ring alters the cellular cortex to form a cleavage furrow and midbody, which serves as a platform for the gathering of the abscission apparatus that governs the final separation of daughter cells [40]. The midbody, initially described by Walther Flemming in the 19th century, forms from the midzone—a bipolar microtubule array that assembles between separating sister chromatids during anaphase [41]. Recently, it has become clear that the midbody acts as a polarity cue during spindle orientation, asymmetric cell division, and cell polarization by orchestrating vesicular transport, cytoskeletal organization, and localized cortical cues [41–44].

Although the overall program of cytokinesis is well-documented, many questions remain, particularly at the molecular level, in part because of the high spatiotemporal complexity of cytokinesis accompanied by conditions for significant force during cleavage [40, 45, 46]. We demonstrated here that Nup58 depletion changed midbody widths. This may arise from the role of Nup58 in bundling and anchoring microtubules at the center of the midzone. Our finding that Nup58 depletion induces cells to be stuck in abscission for hours also raises the idea that Nup58 may contribute to effectual abscission during cytokinesis.

Previously, we reported that Nup62 knockdown induced significantly higher numbers of multipolar spindles compared with controls [28]. In contrast, our present study revealed that Nup58 depletion primarily enhanced monopolar spindle formation. Our live-cell imaging also revealed that Nup58-depleted monopolar spindle cells induce mitotic catastrophe, aneuploidy, and eventually cell death; as these delayed processes (~ 300 min) happened before cytokinesis, midbody formations were often not found (Fig. 5d, e, Additional file 7: Video 5). In mammals, aneuploidy has been linked to cancer progression, which, like cancer development, is a complex process involving functional and genetic abnormalities [27]. Moreover, Nup58 aids metastasis and EMT in lung cancer [37].

Another attractive option is that Nup58 stabilizes microtubules in a specific arrangement to enable the formation of dark zone and/or flanking regions with other midbody proteins (such as KIF4, PLK1, or ESCRT). In a previous study, we reported that Nup62 localized on the mitotic spindles and centrosomes during cell division [28]. In line with this observation, the centrosome has been shown to contribute to abscission and many centrosomal proteins have been found to localize to the midbody ring. Indeed, Nup62 also transiently localizes to the midbody ring at the end of abscission, whereby it interacts with the filamentous actin-capping protein CapG [47]. This study is consistent with our result (Fig. 3a).

Assuming that such a Nup58-Nup62-Nup54 protein subcomplex is required for the correct progression of cytokinesis, then a missing partner might dissolve the complex and result in cytokinetic defects, as observed with Nup58 depletion (Fig. 5h, Additional file 6: Video 4). Given this perspective, we postulate that Nup54 may also localize to centrosomes or midbodies during mitosis. Several organelles, such as the mitotic spindle, centrosome, and midbody, use microtubules as a structural constituent. Interactions between microtubule-dependent organelles and the actin cytoskeleton can be categorized as regulatory or structural [47]. However, the nature of regulatory effects elicited by Nup58 and Nup62 on centrosomes and midbodies is complex, and remains to be resolved in future studies. Indeed, future experiments should also examine the detailed interplay between molecular mechanisms of Nup58, Nup62, Nup54, and other midbody protein interactions.

## Conclusions

We revealed the presence of Nup58 at the centrosome during metaphase and its localization in the midbody during cytokinesis. We propose that mitotic Nup58 localization favors abscission during cytokinesis.

## Methods

### Antibodies

Primary antibodies used for western blot analysis, immunostaining, and immunoprecipitation (IP) were as follows: anti-Nup58 (R36031, Atlas Antibodies, Bromma, Sweden), anti-SAS-6 (sc-82360, Santa Cruz Biotechnology, Dallas, TX, USA), anti-α-tubulin (T9026, clone DM1A, Sigma-Aldrich, St. Louis, MO, USA), anti-γ-tubulin (T5326, clone GTU-88, Sigma-Aldrich), anti-KIF4 (K1765, clone 3E2, Sigma-Aldrich), anti-acetylated α-tubulin (ab24610, clone 6-11B-1, Abcam, Cambridge, UK), anti-Nup62 (N1163, Sigma-Aldrich), anti-Tpr (sc-101294, Santa Cruz Biotechnology), anti-ninein (sc-376420, Santa Cruz Biotechnology), anti Aurora B (ab2254, Abcam), anti-β-actin (sc-47778, Santa Cruz Biotechnology), and GFP tag polyclonal antibody (A11122, Thermofisher, Rockford, IL, USA). Secondary antibodies (Alexa Fluor- or rhodamine-conjugated) were from Molecular Probes (Life Technologies, Carlsbad, CA, USA).

### Cell culture

HeLa cells were obtained from American Type Culture Collection (Manassas, VA, USA). Cell lines were propagated in Dulbecco’s Modified Eagle’s Medium (Nacalai Tesque, Kyoto, Japan) supplemented with 10% (v/v) fetal bovine serum (Life Technologies) and 50 U/ml penicillin–streptomycin (Nacalai Tesque). HeLa cells were maintained in a humidified incubator at 37 °C with 5% CO_2_ [23]. The HeLa EGFP-α-tubulin cell line, used in live cell imaging, was generated by transfection of EGFP-α-tubulin cDNA to HeLa cells and maintained in G418 (600 µg/ml).

### Immunostaining

Procedures used for immunostaining was described previously [23]. Briefly, M-phase-synchronized HeLa cells cultured on coverslips were washed in phosphate-buffered saline (PBS) and fixed for 10 min in ice-cold methanol absolute. Cells were then permeabilized with 0.3% Triton X-100 in phosphate-buffered saline for 10 min at room temperature. After incubation with primary and secondary antibody, samples were mounted onto coverslips with ProLong Gold Antifade reagent (Invitrogen) and were examined on an Olympus FV10i-LIV laser-scanning confocal microscope with a 60× PlanApo/1.45NA DIC objective (Olympus, Tokyo, Japan).

### Immunoprecipitation

Procedures used for IP and western blotting were previously described [23, 26]. Briefly, Mitotic HeLa cells were collected, washed with PBS, spun at 400×*g* for 10 min, and lysed in 1 ml of cold lysis buffer (50 mM Tris–HCl (pH 7.2), 250 mM NaCl, 0.1% Nonidet P-40, 2 mM EDTA, 10% glycerol) containing 1× protease inhibitor mixture (Roche Applied Science) and 1 mM phenylmethylsulfonyl fluoride. Lysates were centrifuged for 30 min at 4 °C at 14,000×*g*. The resulting lysate supernatants were precleared with 50 μl of protein A/G bead slurry (Santa Cruz Biotechnology), mixed with 10 μl of various antibodies as specified, and incubated for 1 h at 4 °C with rocking. The beads were then washed five times with 500 μl of lysis buffer. After the last wash, 50 μl of 1× SDS-PAGE blue loading buffer (New England Biolabs) was added to the bead pellet before loading. Signals were detected with an enhanced chemiluminescence system (GE Healthcare, Chicago, IL) and quantified using a LAS-4000 image analyzer (Fuji Film, Tokyo, Japan) according to the manufacturer’s specifications.

### Midbody isolation

Midbodies were isolated from HeLa cells as described previously [48, 49]. Briefly, HeLa cells were collected after synchronized by thymidine/nocodazole followed by mitotic shake-off. Midbodies were isolated in a buffer (2 mM PIPES, pH 6.9, 0.25% Triton X-100, and 20 µg/ml Taxol) supplemented with protease inhibitor mixture (Roche Applied Science). The final midbody pellet was chilled on ice, washed, and subjected to centrifugation through a cushion of 40% glycerol.

### Plasmids, RNA interference, and transfection

An expression plasmid containing Nup58-GFP was generated by inserting 1800 bp of the Nup58 isoform A gene into the pEGFP-N1 vector (Takara, Kusatsu, Japan). The construct was confirmed by DNA sequencing (PRISM3100-AvantGenetic Analyzer, Applied Biosystems, Foster City, CA, USA). Small interfering (si)RNA duplexes targeting Nup58-specific siRNA (sc-75984) and control siRNA (sc-37007) were purchased from Santa Cruz Biotechnology. Procedures used for transfection was previously described [26].

### Confocal live-cell imaging

Time-lapse analysis of Nup58 dynamics during the metaphase–cytokinesis transition in live cells was recorded from α-tubulin-GFP-, Nup58-GFP- and GFP vector-expressing HeLa cells. Cells were placed into glass-bottomed dished on the stage of an Olympus FV10i-LIV laser-scanning confocal microscope with a 60× PlanApo/1.45NA DIC objective.

### Confocal microscopy image processing

Confocal microscopy images were processed using CellSens (ver.2.1) software (Olympus) to produce deconvoluted confocal images, which were then converted to 16-bit images for processing with Adobe Photoshop CS6 and ImageJ software (imagej.nih.gov/ij/). Procedures used for immunostaining and image processing were previously described [50–52].

### STED nanoscopy, image deconvolution, and analysis

For STED, cells were imaged with a 100× NA 1.4 oil objective on a Leica SP8 gated STED microscope (Wetzlar, Germany). Alexa Fluor 488-labeled probes were excited with a 488-nm wavelength of pulsed white light (WL) using an 80-MHz laser and subsequently depleted with a CW 592-nm STED laser with a typical maximum power of 260–300 mW at the back aperture of the objective (corresponding to ~ 150 MW/cm2 in the focal plane). The settings optimized for a maximum gain in lateral resolution, corresponding to full laser depletion on an internal Leica GaAsP HyD hybrid detector with a time gate of 1.5 ≤ tg ≤ 6.5 ns.

Deconvolution of STED data was carried out using the STED module in Huygens Professional Deconvolution software (version 14.10; Scientific Volume Imaging, Hilversun, the Netherlands), which contains a theoretical estimation of the STED PSF based on values calculated from metadata of the acquired image. For deconvoluted images presented here, we used the calculated Huygens default deconvolution settings that were estimated from the metadata with the exception of a STED immunity fraction of 5%.

### cDNA preparation and quantitative real-time RT-PCR assay

Procedures for cDNA preparation and qRT-PCR were performed as previously described [23, 29]. Total RNA was isolated using a NucleoSpin RNA isolation kit (Macherey–Nagel), and then 500 ng total RNA was used for cDNA preparation using a cDNA synthesis kit (ThermoScript RT-PCR system, Takara). Quantitative real time PCR (qPCR) was performed using a Thermal Cycler Dice Real Time System with SYBR Premix Ex Taq II (Takara). The primer sets were as follows: for Nup58, forward: 5′-CACAGCCATCTCTGGGAGTT-3′ and reverse: 5′-GCCAAAGCCTGCACTAAGAC-3′ for Nup62, forward: 5′- ACATCGATGCACAGCTCAAG-3′ and reverse: 5′-ACTGCAGTGAGTCCATGTGC -3′; for SAS-6, forward: 5′-CGCAGGCTGTTTGAAATGTA-3′ and reverse: 5′-TGTATGTGACGCCCATTCAT-3′; for GAPDH, forward: 5′-GTCAGTGGTGGACCTGACCT-3′ and reverse: 5′-AGGGGTTCTACATGGCAACTG-3′. Relative mRNA expression levels of target genes were calculated using *GAPDH* as an internal control.

### Statistics

Statistical analyses were performed using Prism 7 (GraphPad Software, San Diego, CA, USA). Data are presented as mean ± standard deviation (SD). Statistically significant differences in mean or median values between respective groups were tested by a Student’s *t* test or a Mann–Whitney U test. *P* values < 0.05 with a confidence interval of 95% were considered to indicate a statistically significant difference.

## Additional files

**Additional file 1: Figure S1.** Colocalization of Nup58 with midbody protein markers γ-tubulin (a) and KIF4 (b) acquired with confocal microscopy. **Figure S2.** z-plane confocal images of HeLa cells during metaphase and its maximum projections showing colocalization of Nup58 with midbody protein markers α-tubulin (a), γ-tubulin (b) and SAS-6 (c). **Figure S3.** Depletion of Nup58 in HeLa cells. HeLa cells were transfected with control or Nup58 siRNA, then cell lysates were collected 72 h after transfection, analyzed for knockdown efficiency by immunoblot of Nup58 and β-actin expression (upper panel) and for expression of Nup62 by confocal images (lower panel) Green, anti- γ-tubulin; red, anti-Nup62; blue, chromatin (DAPI). Scale bars, 5 µm. **Figure S4.** Expression of importin-β after depletion of Nup58 in HeLa cells.

**Additional file 2: Video 1.** Localization of Nup58 on the centrosome in Nup58–GFP-expressing HeLa cells (right) during mitosis compared with GFP-vector-expressing HeLa cells (left) acquired with live-cell imaging

**Additional file 3: Video 2.** Live-cell imaging of GFP-vector-expressing HeLa cells during cytokinesis from midbody maturation to final abscission.

**Additional file 4: Video 3a.** Localization of Nup58 on the midbody in Nup58–GFP-expressing HeLa cells during cytokinesis from midbody maturation to final abscission acquired with live-cell imaging.

**Additional file 5: Video 3b.** Magnification of Nup58–GFP-expressing HeLa cells from video 3a. The inset shows enlarged detail of midbody areas in observed cells. Arrowheads indicate HeLa cell midbodies during cytokinesis.

**Additional file 6: Video 4.** Delayed cytokinesis of Nup58 siRNA transfected GFP-α-tubulin expressing HeLa cells (right) compared with the control (left) acquired with live-cell imaging.

**Additional file 7: Video 5.** A halt in cell division of Nup58 siRNA transfected GFP-α-tubulin expressing HeLa cells (right) compared with the control (left) acquired with live-cell imaging.

## Data Availability

All data generated or analyzed during this study are included in the article (and its additional files).
