# Supplementary figures and images for: Nucleoporin Nup58 localizes to centrosomes and mid-bodies during mitosis
Source: Cell Div. 2019 Aug 3;14:7. doi: 10.1186/s13008-019-0050-z (PMC6679547; doi:10.1186/s13008-019-0050-z)

## Supplementary Figure 1

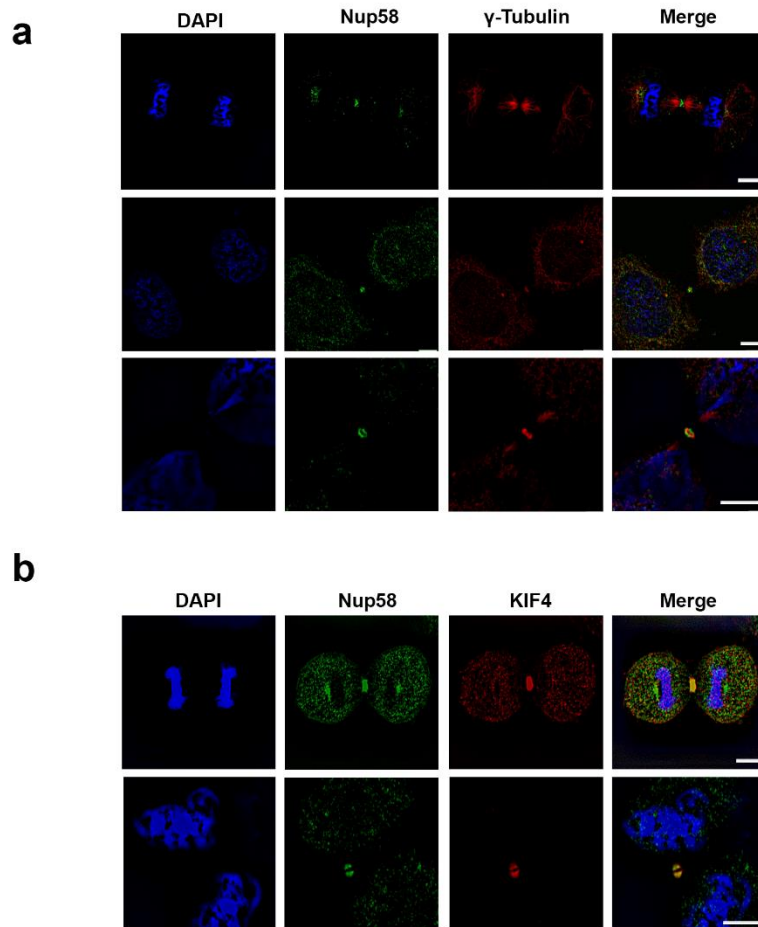

Supplementary Figure 2

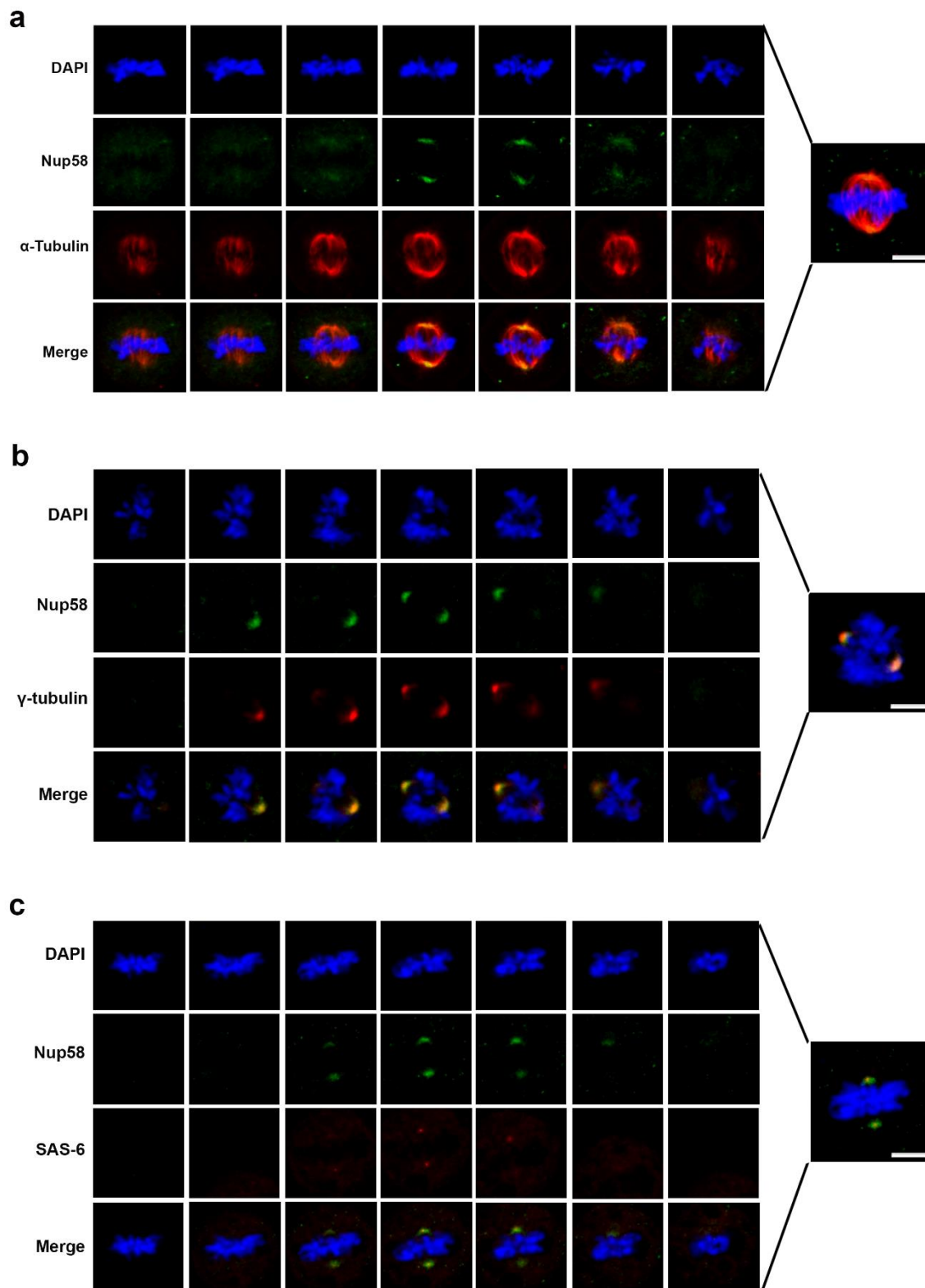

Supplementary Figure 3

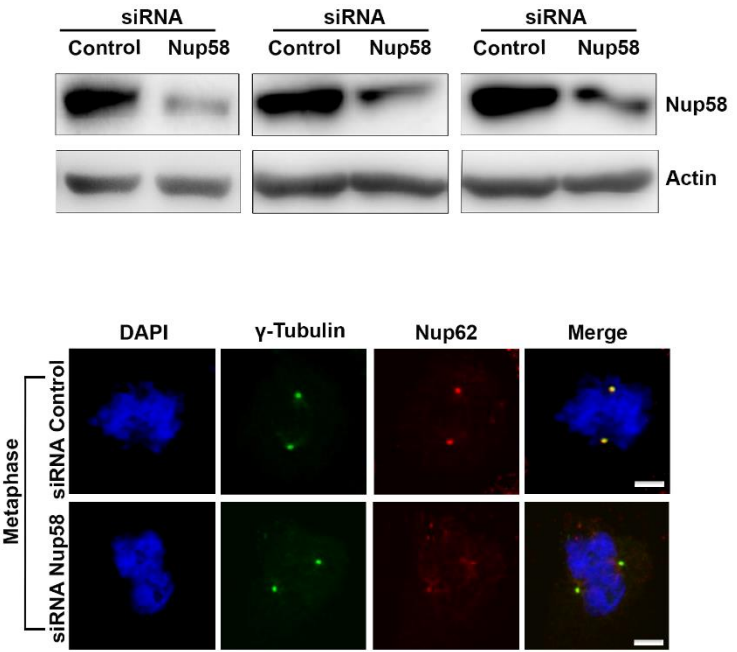

Supplementary Figure 4

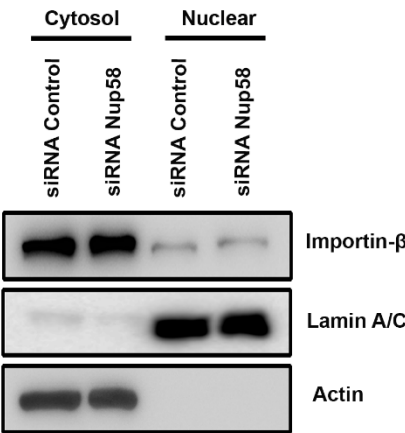

Supplement: Supplementary file 1 — Additional file 1: Figure S1. Colocalization of Nup58 with midbody protein markers γ-tubulin (a) and KIF4 (b) acquired with confocal microscopy. Figure S2. z-plane confocal images of HeLa cells during metaphase and its maximum projections showing colocalization of Nup58 with midbody protein markers α-tubulin (a), γ-tubulin (b) and SAS-6 (c). Figure S3. Depletion of Nup58 in HeLa cells. HeLa cells were transfected with control or Nup58 siRNA, then cell lysates were collected 72 h after transfection, analyzed for knockdown efficiency by immunoblot of Nup58 and β-actin expression (upper panel) and for expression of Nup62 by confocal images (lower panel) Green, anti- γ-tubulin; red, anti-Nup62; blue, chromatin (DAPI). Scale bars, 5 µm. Figure S4. Expression of importin-β after depletion of Nup58 in HeLa cells. [file 13008_2019_50_MOESM1_ESM.pdf]
